# Hybrid quantum annealing via molecular dynamics

**DOI:** 10.1038/s41598-021-87676-z

**Published:** 2021-04-19

**Authors:** Hirotaka Irie, Haozhao Liang, Takumi Doi, Shinya Gongyo, Tetsuo Hatsuda

**Affiliations:** 1 AI R&I Division, Advanced Research and Innovation Center, DENSO CORPORATION, Global R & D Tokyo, Tokyo, 108-0075 Japan; 2 RIKEN Interdisciplinary Theoretical and Mathematical Sciences Program (iTHEMS), Saitama, 351-0198 Japan; 3 RIKEN Nishina Center (RNC), Saitama, 351-0198 Japan; 4 Department of Physics, Graduate School of Science, The University of Tokyo, Tokyo, 113-0033 Japan

**Keywords:** Computational science, Information theory and computation, Theoretical physics

## Abstract

A novel quantum–classical hybrid scheme is proposed to efficiently solve large-scale combinatorial optimization problems. The key concept is to introduce a Hamiltonian dynamics of the classical flux variables associated with the quantum spins of the transverse-field Ising model. Molecular dynamics of the classical fluxes can be used as a powerful preconditioner to sort out the frozen and ambivalent spins for quantum annealers. The performance and accuracy of our smooth hybridization in comparison to the standard classical algorithms (the tabu search and the simulated annealing) are demonstrated by employing the MAX-CUT and Ising spin-glass problems.

## Introduction

Combinatorial optimizations are ubiquitous and generally represented by the Ising spin-glass model, which is computationally classified as an NP-hard problem^1^. The quantum annealing with a transverse-field Ising model^2,3^ as well as the adiabatic quantum computation^4,5^ provide metaheuristic quantum algorithms for such difficult combinatorial optimizations. They utilize adiabatic evolution of quantum bits (qubits) to find the ground state of Ising spin-glass models. Since quantum-annealing processors (quantum annealers) have become available^6^, practical usage as well as fundamental researches on quantum optimization has largely been developed in recent years (see e.g. Refs.^7,8^ and references therein).

Despite the great progress that has been taken place in the development of quantum optimization, the number of qubits as well as the noise control are still limited. To ulilize such noisy intermediate-scale quantum (NISQ) devices^9^, hybrid systems that are capable of dealing with large-scale optimization problems while using relatively small quantum optimization need to be developed. So far, various hybrid algorithms have been proposed in the literature (see, e.g. Refs.^10–18^ and references therein). Most of them are based on the idea of decomposing original large-scale problem into subproblems to be treated by available quantum devices, so that multiple iterations between classical and quantum solvers are required, while some are based on identification of a plausible subproblem by fixing persistent variables in multiple sampling of classical solvers^17,18^.

**Figure 1:**
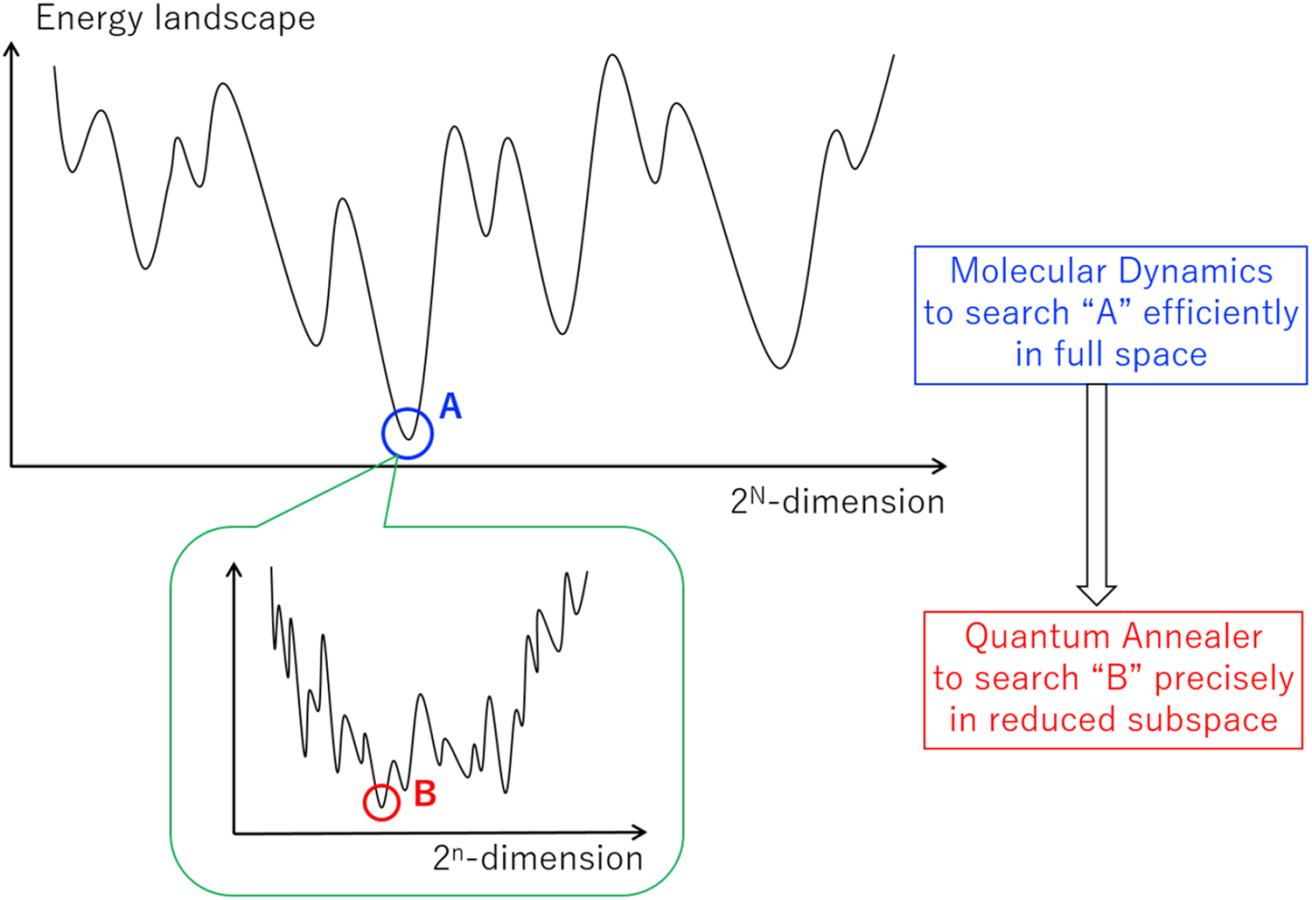
Concept of hybrid quantum annealing via molecular dynamics.

In this paper, we propose a novel hybrid system of quantum optimization, Hybrid Quantum Annealing via Molecular Dynamics (HQA-MD, or HQA for short), based on a combination of the classical molecular dynamics (MD)^19^ and the quantum annealing (QA)^2^. The concept of HQA is illustrated in Fig. 1. Consider the Ising spin-glass with *N* number of sites. Only a single run of the classical MD solver with continuous flux variables is capable of identifying a set of low-energy spin configurations with $2^n$-dimension indicated schematically by the region *A* in the full $2^N$-dimensional space, where *n* is an arbitrarily chosen number smaller than *N*. The quantum solver with quantum spin variables can then resolve the fine structure of the reduced $2^n$-dimensional subspace to find the minimum *B*. Thus, our classical MD solver acts as a powerful preconditioner to extract difficult spin variables automatically from the huge energy landscape and send them to the quantum annealer.

For HQA to work in practice, it is crucial to develop suitable classical Hamiltonian dynamics. The molecular dynamics of the classical fluxes can be used as a powerful preconditioner to sort out the “frozen” and “ambivalent” spins for quantum annealers, as we see below. Since both classical and quantum Hamiltonians have the same roots, HQA constitutes a seamless scheme for quantum–classical hybridization, so that some of the various developments that improve the performance of QA can also be imported into HQA and be utilized in large-scale Ising spin-glass models. We note that the classical part of our HQA has some similarity with SVMC (Spin-vector Monte Carlo)^20^, CIM (coherent Ising machine)^21,22^, and SBM (simulated bifurcation machine)^23^. Therefore, it is natural to expect that our hybrid scheme can be also applied to such continuous optimizations schemes.

## Results

A large class of combinatorial optimization problems can be mapped onto the Ising model

1 $$\begin{aligned} {\mathcal{H}}_{\mathrm{Ising}} (s)= \frac{1}{2} \sum _{i\ne j}^N J_{ij} s_i s_j + \sum _{i=1}^N h_i s_i, \end{aligned}$$

with the Ising variables ($\{ s_i = \pm 1 \}_{i=1}^N$), the symmetric coupling ($J_{ij}$) and the external field ($h_i$)^24^. The quantum annealing (QA) of transverse-field Ising model^2^ provides an efficient method to solve the ground state of the system through the quantum deformation of ${\mathcal{H}}_{\mathrm{Ising}}$ as

2 $$\begin{aligned} {\mathcal {H}}_{\mathrm{QA}}(\sigma ;\tau ) =A(\tau ) \biggl [ - \sum _{i=1}^N \sigma _i^x \biggr ] + B(\tau ) \biggl [ \frac{1}{2} \sum _{i\ne j}^N J_{ij} \sigma _i^{z} \sigma _j^{z} + \sum _{i=1}^N h_i \sigma _i^z \biggr ], \end{aligned}$$

where $\sigma _i^x, \sigma _i^z$ (and also $\sigma _i^y$) are 2$\times$2 Pauli matrices at each site *i* (= $1, 2, \dots , N)$, and $\tau$ is a fictitious time taken to be in an interval [0, 1]. The scheduling functions $A(\tau )$ and $B(\tau )$ are chosen so that ${\mathcal{H}}_{\mathrm{QA}}(\tau )$ interpolates adiabatically the non-interacting spins with transverse field at initial time ($A(0) \gg B(0)$ ) and the classical Ising spin-glass at final time ($A(1)\ll B(1)$). (The actual scheduling functions in our numerical experiments below are $A=A_{\mathrm{DW}}/2$ and $B=B_{\mathrm{DW}}/2$ where $A_{\mathrm{DW}}$ and $B_{\mathrm{DW}}$ are the scheduling functions given in Fig. 2 of Ref.^25^.) In the actual quantum annealing devices, quantum Ising spin is realized by the superconducting flux qubits described by a quantum Hamiltonian ${\mathcal {H}}_{\mathrm{device}}(\hat{\varphi }, \hat{p} ; \tau )$ written by the flux operators $\hat{\varphi }_i$ and their conjugates $\hat{p}_i$ with the canonical commutation, $[\hat{\varphi }_j, \hat{p}_k ] = i \hbar \delta _{jk}$ (see e.g. Ref.^26^).

## Molecular dynamics for flux variables

**Figure 2:**
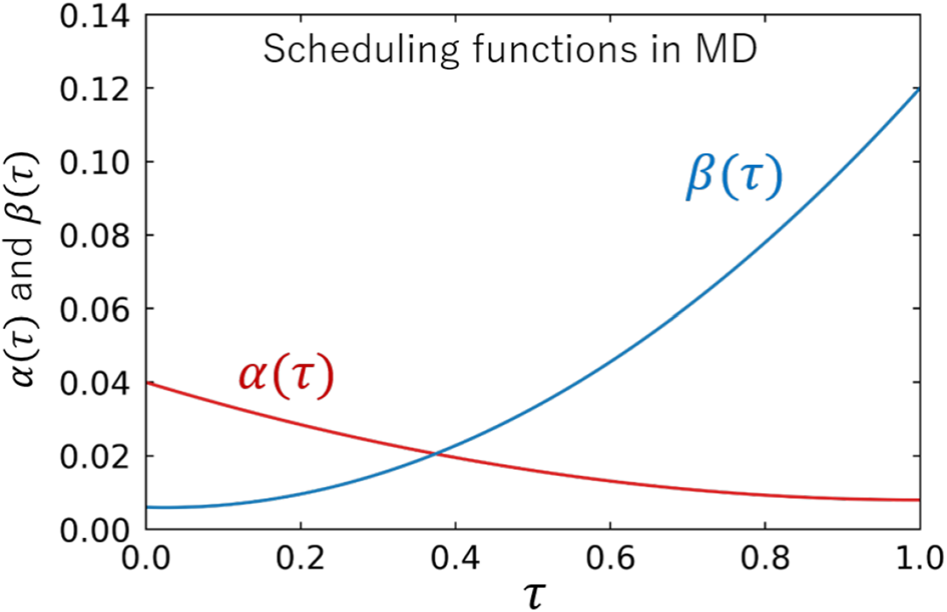
The actual scheduling functions in our MD run. See “Methods” section for their analytic forms.

To construct a seamless hybrid between quantum and classical solvers, we introduce a classical Hamiltonian for flux variables:

3 $$\begin{aligned} {\mathcal {H}}_{\mathrm{MD}}(\varphi , p; \tau ) = \alpha (\tau ) \sum _{i=1}^N \Bigl ( \frac{p_i^2}{2} + V(\varphi _i) \Bigr ) + \beta (\tau ) \biggl [ \frac{1}{2} \sum _{i\ne j}^N J_{ij} \varphi _i \varphi _j + \sum _{i=1}^N h_i |\varphi _i| \varphi _i \biggr ] , \end{aligned}$$

where “MD” stands for the Molecular Dynamics, $\{\varphi _i\}_{i=1}^N$ ($\{p_i\}_{i=1}^N$) are the continuous flux variables (continuous conjugate momenta) which are classical counter parts of $\{\hat{\varphi }_i\}_{i=1}^N$ ($\{ \hat{p}_i\}_{i=1}^N$). The MD evolution is parametrized by $\tau =t/t_f \in [0,1]$ with $t \in [0, t_f]$ being the actual evolution time. The potential term $V(\varphi )$ is a convex downward function of the form $V(\varphi )= \varphi ^M$ $(M=4, 6, 8, \dots )$. Shown in Fig. 2 are the actual scheduling functions ($\alpha (\tau )$ and $\beta (\tau )$) to be used in the present paper. The analytic forms are given in “Methods” section.

It is in order here to discuss the basic properties of the above classical Hamiltonian: The term proportional to $\alpha (\tau )$ in Eq. (3) ensures that each classical flux variable oscillates around $\varphi _i =0$ in early times. It plays a similar role as the transverse-field term proportional to $A(\tau )$ in Eq. (2) which drives each spin state in early times to be an equal superposition of up and down. The term proportional to $\beta (\tau )$ in Eq. (3) is a direct analogue of the Ising model: By decomposing the flux variables as $\varphi _i = |\varphi _i| {\mathrm{sgn}} ({\varphi }_i)$, one finds the “correspondence” between the terms in Eqs. (2) and (3); $B J_{ij} \leftrightarrow \beta J_{ij} |\varphi _i \varphi _j |$ and $B h_{i} \leftrightarrow \beta h_{i} |\varphi _i \varphi _i|$. We note that the classical dynamical system achieves a faithful representation of the Ising model, only when all $|\varphi _i|$ are frozen to a positive constant $\mu$ and the equality $B=\beta \mu ^2$ gets satisfied. However, this cannot be achieved even for ideal MD solvers, and this is a generic problem of all classical solvers using continuous dynamical systems. On the other hand, our MD solver plays a role of a preconditioner for the quantum annealing, so that $\varphi _i$’s need not to settle down to $\pm \mu$. This is also the reason why $\alpha (\tau =1)$ can be non-zero as shown in Fig. 2.

The Hamilton equations for the time evolution of the flux variables reads

4 $$\begin{aligned} g \, \frac{d \varphi _i }{d\tau }= \frac{\partial {\mathcal {H}}_{\mathrm{MD}}(\varphi , p;\tau )}{\partial p_i},\qquad g \, \frac{d p_i }{d\tau } = - \frac{\partial {\mathcal {H}}_{\mathrm{MD}}(\varphi , p ;\tau )}{\partial \varphi _i}, \end{aligned}$$

where $\tau = t/t_f \equiv gt$. The motion of the flux variables becomes adiabatic for $g \rightarrow 0$. We solve the above equations by the leapfrog algorithm (“Methods” section) on a GPGPU machine. As the initial conditions, we take $\varphi _{i}(\tau =0)=0$, with $p_i(\tau =0)$ randomly chosen to be $+1$ or $-1$. As for the convex potential, we have tested $M=4, 6, 8$ and found that $M=6$ shows the best performance in terms of the evolution time and the achieved accuracy, so that we use this value throughout this paper.

**Figure 3:**
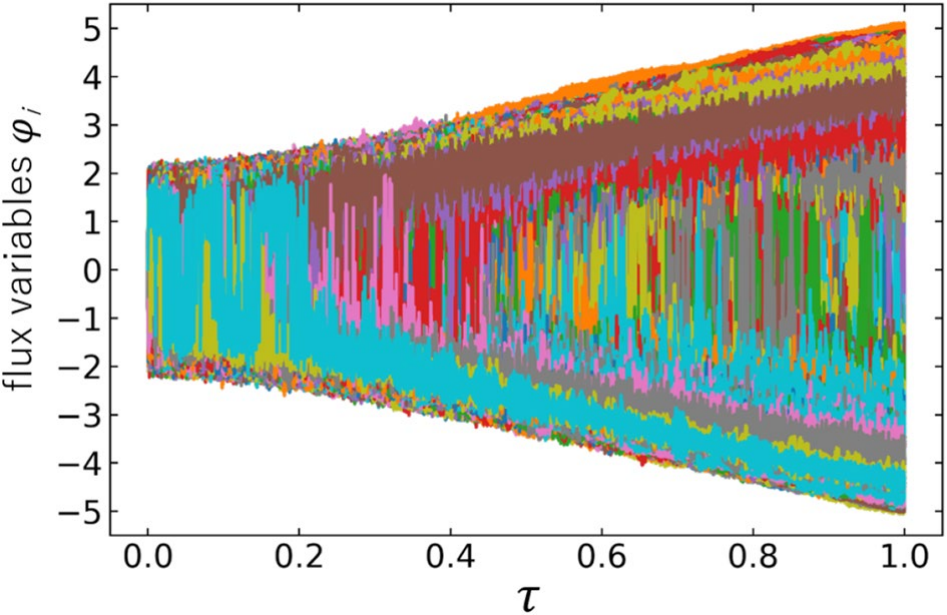
Trajectories of all flux variables $\{\varphi _i \}_{i=1}^N$ for a typical Ising spin-glass model with $N=10,000$ and $(\delta \tau )^{-1}= 50{,}000$.

**Figure 4:**
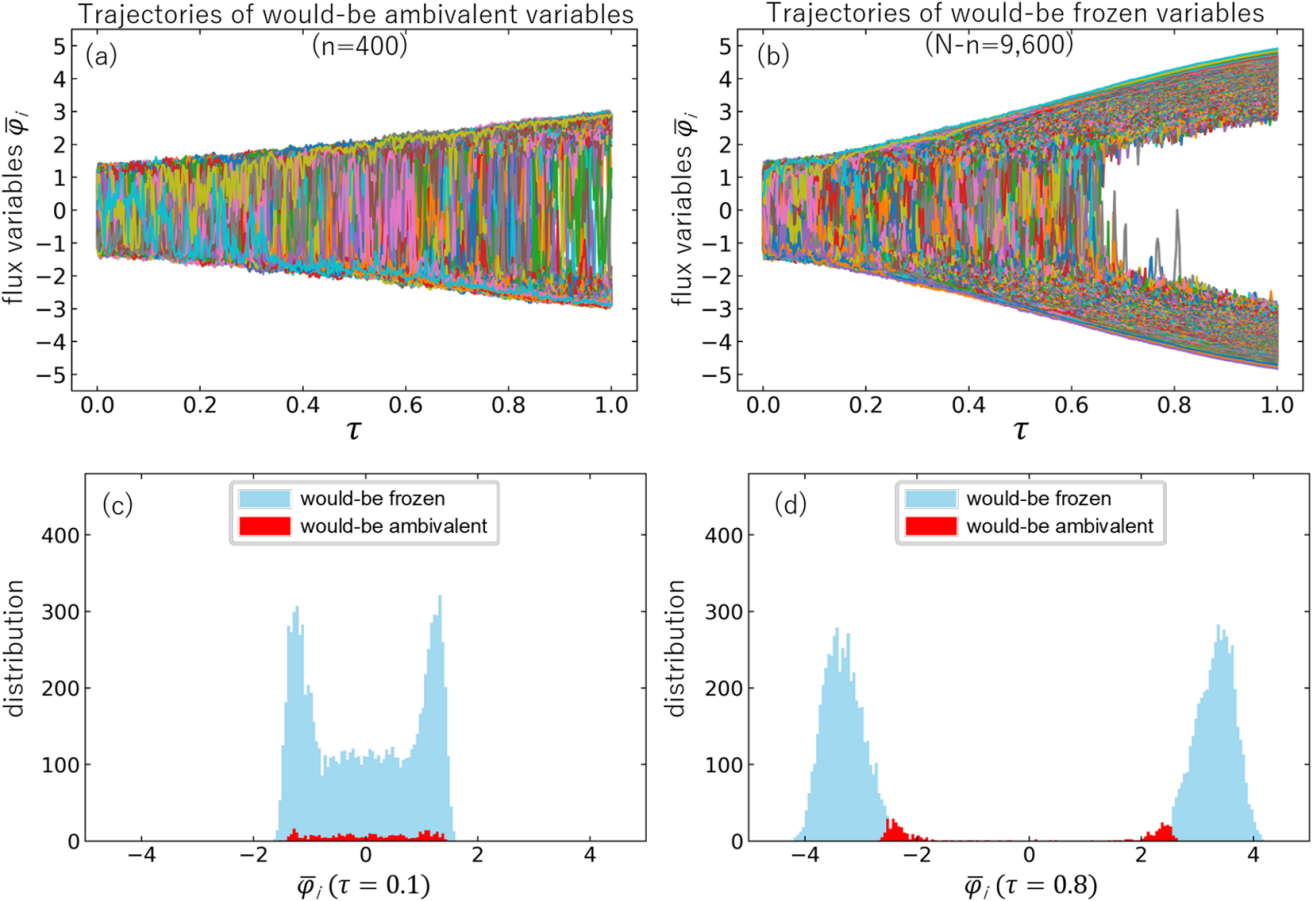
(**a**) Trajectories of would-be ambivalent variables $\overline{\varphi }_{i^{\prime}}$ with $i^{\prime}=1,2, \dots , n$. (**b**) Trajectories of would-be frozen variables $\overline{\varphi }_{i^{\prime}}$ with $i^{\prime}=n+1, \dots , N$. Here, *n* and *N* are taken to be 400 and 10,000, respectively. Distributions of would-be frozen and ambivalent variables at $\tau =0.1$ (**c**) and at $\tau =0.8$ (**d**).

## Sorting frozen and ambivalent variables

Shown in Fig. 3 are all trajectories $\{ \varphi _i \}_{i=1}^N$ ($N=10{,}000$) as a function of $\tau$ in a test MD evolution with a single set of Ising spin-glass parameters picked up randomly in the intervals, $-1 \le J_{ij} \le +1$ and $-2 \le h_i \le +2$. The MD time step $\delta \tau$ is chosen to be 1/50,000. Moreover, we make an identification, $g=\delta \tau$, so that the small time step corresponds to the adiabatic evolution. Although there is a tendency that $\varphi _i$ fall into two categories with positive sign and negative sign, we need a criterion to separate them in a quantitative manner. For that purpose, let us introduce time-averaged flux variables,

5 $$\begin{aligned} \overline{\varphi }_i(\tau ) \equiv \frac{1}{\delta } \int ^{\tau }_{\tau -\delta } d\tau ^{\prime} \, \varphi _i(\tau ^{\prime}), \end{aligned}$$

where the interval $\delta$ should be sufficiently larger than $\delta \tau$ and sufficiently smaller than 1. Then all trajectories can be sorted by using their magnitudes at $\tau =1$ as $\bigl | \overline{\varphi }_{1^{\prime}} (\tau =1) \bigr | \le \bigl | \overline{\varphi }_{2^{\prime}} (\tau =1) \bigr | \le \cdots \le \bigl | \overline{\varphi }_{n^{\prime}} (\tau =1 ) \bigr | \le \cdots \le \bigl | \overline{\varphi }_{N^{\prime}} (\tau =1 ) \bigr |$ where $i^{\prime}$ is an index after the sorting and *n* is an arbitrarily chosen integer less than *N*. Then, we call the *n* low-lying trajectories, $\left\{ \varphi _{i^{\prime}}\right\} _{i^{\prime}=1}^n$, as *ambivalent* variables, and the rest is called *frozen* variables. Shown in Fig. 4a with $\delta = 100 \cdot \delta \tau$ are the time-averaged trajectories $\left\{ \overline{\varphi }_{i^{\prime}} (\tau ) \right\} _{i^{\prime}=1}^n$ with $n=400$, while Fig. 4b shows all the other 9600 trajectories. These figures indicate that most of the flux variables are frozen in sign after the MD evolution, while small number of ambivalent variables remains at $\tau =1$. In Fig. 4c,d, we show the distributions of the would-be frozen and ambivalent variables at an early time ($\tau =0.1$) and at a late time ($\tau =0.8$). As the time goes by, the distinction between two categories becomes prominent.

**Figure 5:**
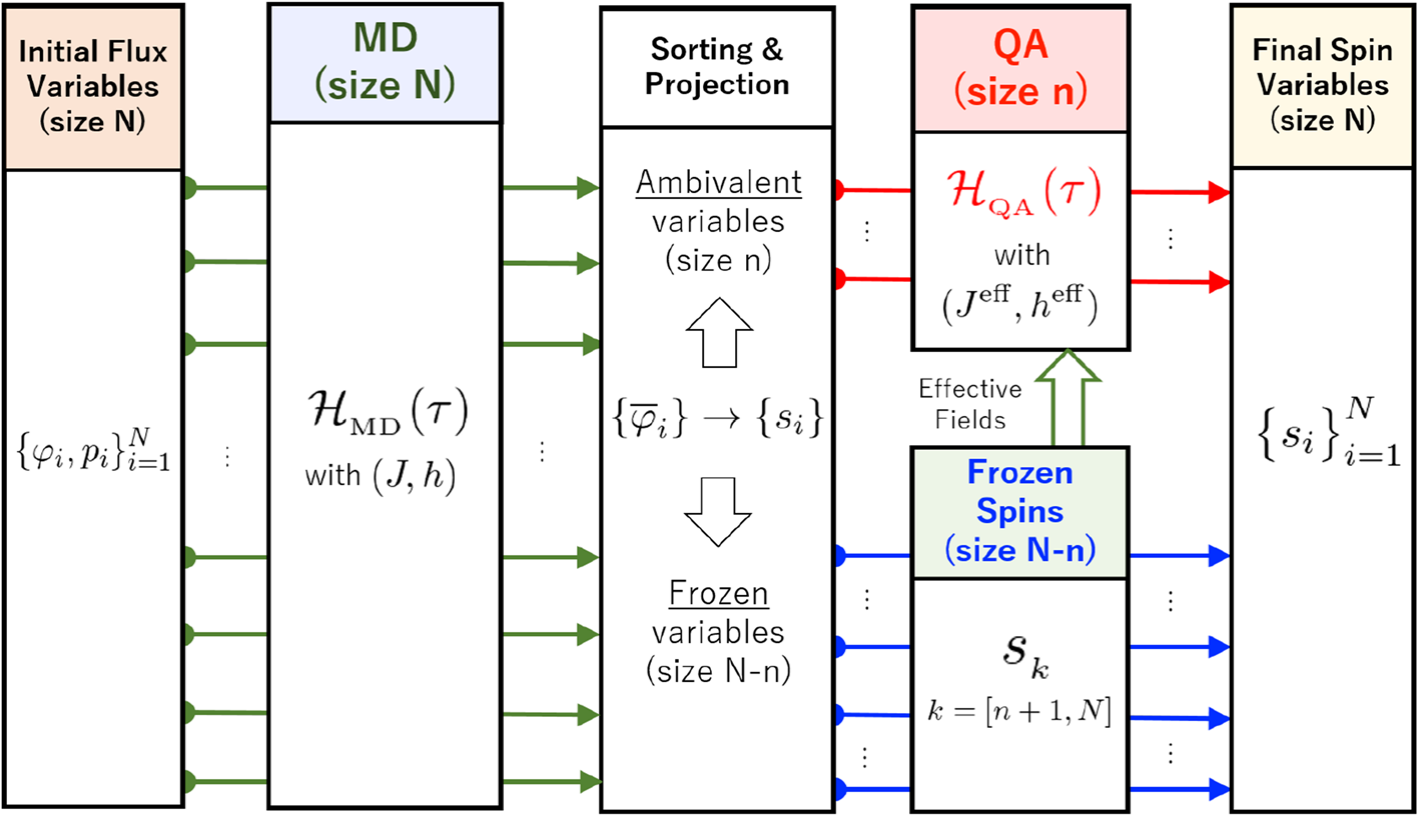
Flowchart of the hybrid quantum annealing (HQA) via molecular dynamics (MD).

## Hybrid quantum annealing via molecular dynamics

**Figure 6:**
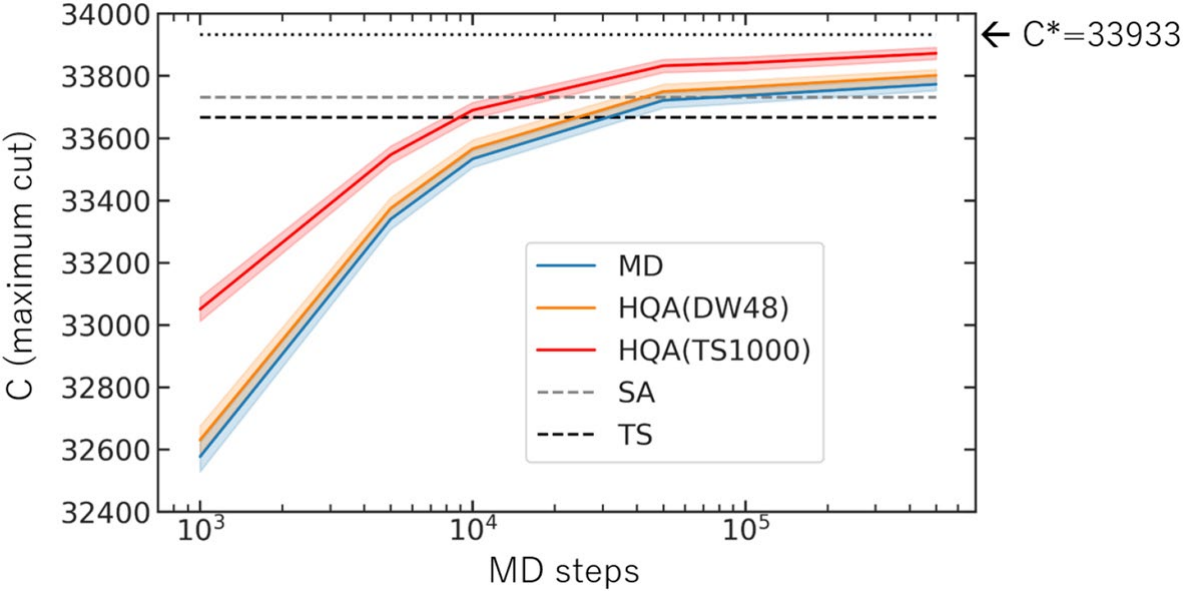
The maximum cut *C* in the MAX-CUT problem on a complete graph with 2000-node ($K_{2000}$) obtained by different solvers. Theoretical estimate of the maximum cut is $C^*=33{,}933$.

Our MD evolution combined with the above sorting algorithm can extract the ambivalent variables efficiently. For instance, as is shown in Fig. 6 (the line labeled by “MD”), the obtained configurations successfully approach to the optimum. However, it takes an exponential time for those ambivalent variables to really settle down, and it is not guaranteed to converge to the optimum. Therefore, it is highly impractical to continue the MD evolution toward $\alpha =0$. Our approach to circumvent this issue is a novel hybrid scheme (HQA) where MD is used as a powerful preconditioner for QA. Currently available quantum annealers as well as quantum hybrid solvers are still limited in size and accuracy. Nevertheless, as will be demonstrated below, the HQA complements the large-scale capability of such quantum solvers and enhances the performance in solving large *N* optimization problems.

Our HQA is operated in the following way: We fix the frozen spins ($k^{\prime} =n+1, \dots , N$) by the projection $s_{k^{\prime}} = {{{\mathrm{sgn}}\,}}\bigl ( \overline{\varphi }_{k^{\prime}} (\tau =1) \bigr )$, while the ambivalent spins ($i^{\prime} =1, \dots , n$) are sent to a reduced size Ising subsystem with the Hamiltonian,

6 $$\begin{aligned} {{\mathcal {H}}^{\prime}}_{\text {Ising}}(s) = \frac{1}{2} \sum _{i^{\prime} \ne j^{\prime}} ^{n} J^{\mathrm{eff}} _{i^{\prime}j^{\prime}} s_{i^{\prime}} s_{j^{\prime}} + \sum _{i^{\prime}=1}^n h^{\mathrm{eff}}_{i^{\prime}} s_{i^{\prime}} \equiv {{\mathcal {H}}}_{\text {Ising}}(s|s_{k^{\prime}=n+1, \dots , N}:{\mathrm{frozen}} ) - ({\mathrm{const}}.). \end{aligned}$$

Here the effective couplings read

7 $$\begin{aligned} J^{\mathrm{eff}}_{i^{\prime}j^{\prime}} = J_{i^{\prime}j^{\prime}}, \ \ \ h^{\mathrm{eff}} _{i^{\prime}} = h_{i^{\prime}} + \sum _{k^{\prime}=n+1}^{N} J_{i^{\prime} k^{\prime}} s_{k^{\prime}}, \ \ \bigl (i^{\prime}, j^{\prime} = 1, 2, \dots , n \bigr ) . \end{aligned}$$

This small subsystem of *n* degrees of freedom can be solved by embedding it into a quantum annealer or other Ising solvers. Shown in Fig. 5 is an overall flowchart of our HQA starting from initial flux variables $\{ \varphi _i, p_i \}_{i=1}^N$ and ending with the final Ising-spin variables $\{ s_i \}_{i=1}^N$.

## HQA for MAX-CUT problem

To demonstrate how our HQA works, let us consider the MAX-CUT problem which is to find the size of the maximum cut (*C*) in a given undirected graph. We take an all-to-all connected graph with 2000-node ($K_{2000}$) having the random bimodal edge-weight $w_{ij} = \pm 1$ with zero-mean. This problem has been used for benchmarking of various classical solvers including CIM^21,22^ and SBM^23^. Mapping this problem into the Ising model (“Methods” section) with $J_{ij} = w_{ij}$, $h_i=0$ and $N=2000$, we compare the performance of three different cases; our MD solver alone, HQA(DW48) which is an HQA with the $n=48$ subsystem solved by the D-Wave machine (DW_2000Q_5^25^), and HQA(TS1000) which is an HQA with the $n=1000$ subsystem solved by the classical tabu search implemented as QBSolv^27^.

For reference classical solvers, we consider the simulated annealing (SA) (dwave.neal^28^) and the tabu search (TS). The number of sweeps of SA is set to be $N_{\mathrm{sw}}=$1000 with the inverse temperature $\beta$ increasing geometrically from $\beta _{\mathrm{I}} = 0.01$ to $\beta _{\mathrm{F}} = 1.0$ at every single sweep. These values of $\beta _{{\mathrm{I}}, {\mathrm{F}}}$ are chosen as a result of optimization over the random 100 instances. In our classical computational system (“Methods” section), the computational cost of $N_{\mathrm{sw}} \times N$ ($=1000 \times 2000)$ SA steps is comparable to that of the $10^6$ MD steps. TS in the present study (QBSolv implemented by D-Wave Systems, inc.) is already an optimized Tabu Search compared to a simple Tabu Search algorithm, and the computational time of TS is comparable to (or longer than) SA for more than a few thousands variables.

In Fig. 6, the horizontal axis represents the number of computational steps $(\delta \tau )^{-1}$ in MD, while the vertical axis is the number of maximum cut (*C*) obtained by different solvers. Colored solid curves are the results of different solvers, MD, HQA(DW48) and HQA(TS1000). Note that the figure only shows dependence of MD steps. The band associated with each line represents $\pm 1\sigma$ confidence interval for 100 instances. (In actual numerical experiments, each $J_{ij}$ is combined with a mirror instance $-J_{ij}$ to ensure $C_0 \equiv \frac{1}{4} \sum _{i \ne j} J_{ij} =0$.) In Supplementary Note 1, the initial-condition dependence of MD and HQA is shown with a single instance and 100 initial conditions. Theoretical value of *C* using the finite size scaling analysis in statistical mechanics is $C^* = -E^*/2 \simeq 33{,}933$^29^ (“Methods” section) as shown by the dotted line. Here $E^*$ is the ground-state energy of the Ising model averaged over instances. The results of SA and TS with the above setting are shown by the gray dashed line and the black dashed line, respectively.

From the figure, one finds that the MD alone reaches up to 0.4 $\%$ deviation from $C^*$ after 500, 000 MD steps. This is more accurate than the results of other classical solvers such as SA (0.6% deviation) and TS (0.8% deviation) obtained under the comparable computational time. Moreover, HQA shows further improvement of the solution toward $C^*$. Here with the same 500,000 MD steps, HQA(DW48) and HQA(TS1000) reach up to 0.3$\%$ and 0.2$\%$ accuracy, respectively. Note here that the primary computational time of HQA is consumed by the MD part in our computational systems (“Methods” section). For example, the ratio of the computational time between DW48 and MD (500,000 MD steps) is 0.007, excluding the cloud connection to D-Wave machine. Also, the ratio between TS1000 and MD (500,000 MD steps) is 0.24.

If one continuously proceeds with the classical solvers (such as MD, TS or SA) to achieve the improvement the computational time will grow substantially: For example, to achieve 0.2% accuracy in SA, we find it necessary to increase the number of sweeps 10 times, $N_{\mathrm{sw}}=10{,}000$. Our HQA approach avoids such difficulty by extracting and solving a computationally hard subproblem by quantum annealer or its quantum–classical hybrid systems. It is worth noting that even HQA(DW48) shows the improvement. For such a small system $(n=48)$, quantum annealer is not strong enough to be compared with other classical solvers. However, it is still surprising that such a small subsystem improves the performance.

**Figure 7:**
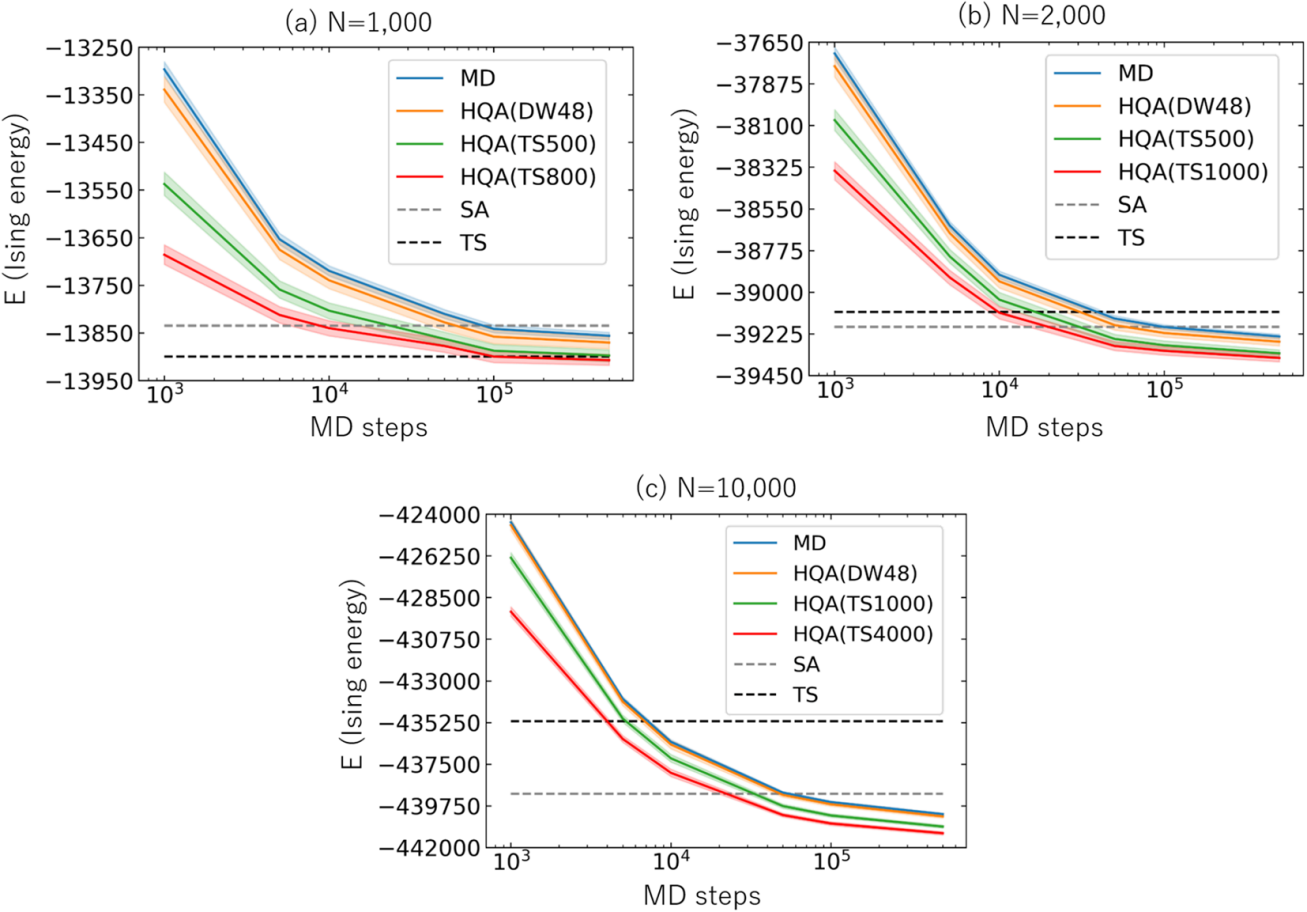
Results of the Ising energy *E* for the Ising spin-glass problem averaged over 100 instances by using different solvers with three different system sizes (**a**) $N=1000$, (**b**) $N=2000$, and (**c**) $N=10{,}000$.

## HQA for Ising spin-glass problem

Finally we consider a general Ising spin-glass model with 100 instances whose parameters $J_{ij}$ and $h_i$ are randomly chosen in the interval $-1 \le J_{ij} \le +1$ and $-2 \le h_i \le +2$ (where the uniform distribution is utilized). Total system sizes are taken to be $N=1000$, 2000, and 10, 000 for several different values of *n* in Fig. 7a,b,c. Results of the Ising energy averaged over instances $E \equiv \left\langle {\mathcal{H}}^{\mathrm{(min)}}_{\mathrm{Ising}}(s) \right\rangle$ are plotted as a function of the MD steps $(\delta \tau )^{-1}$ ranging from 1000 to 500, 000. (See Supplementary Note 2 for the adiabaticity of MD evolution and its relation to the choice of the scheduling functions.) The colored solid curves are obtained by MD, HQA(DW48), HQA(TS500), HQA(TS800), HQA(TS1000) and HQA(TS4000). The band associated with each line represents $\pm 1\sigma$ confidence interval for 100 instances.

The results of reference classical solvers, SA, and TS, are drawn by the gray and black dashed lines, respectively. The number of sweeps of SA is set to be $N_{\mathrm{sw}}=1000$ with the inverse temperature $\beta$ increasing geometrically from $\beta _{\mathrm{I}} = 0.01$ to $\beta _{\mathrm{F}} = 1.0$ at every single sweep. This value of $\beta$ is chosen as a result of optimization over random 100 instances for the Ising spin-glass model. Similar to the MAX-CUT cases, the computational cost of $N_{\mathrm{sw}} \times N\, (=1000 \times 1000 \sim 1000 \times 10{,}000)$ SA steps is comparable to the $10^6\sim 10^7$ MD steps. Note that the computational time of TS is again comparable to (or longer than) SA for more than a few thousands variables.

If the system size *N* exceeds a few thousand, the accuracy of MD becomes better than that of other classical solvers. For example, in the case of $N = 10{,}000$, the precision of SA with $N_{\mathrm{sw}}=1000$ and 10, 000 can be obtained by MD alone with 50,000 and 500,000 MD steps, respectively. Moreover, we find that HQA achieves better accuracy even further than the MD solution, where our MD solver acts as a powerful preconditioner to extract difficult spin variables even in the large-size problems.

## Discussion

In this paper, we introduced a quantum–classical hybrid scheme (HQA-MD, or HQA for short) which utilizes the molecular dynamics as a preconditioner for quantum annealing. By taking a classical Hamiltonian for flux variables associated with spin variables, we have demonstrated that our HQA can solve combinatorial optimization problems with high accuracy. Moreover, our HQA shows better performance as the system size becomes larger. There are various interesting questions to be studied further. Among others, generalization of HQA with non-stoquastic interactions needs to be developed e.g. by adding off-diagonal kinetic terms in the MD solver, $\sum _{i<j} \ell _{ij} \, p_i p_j$^30^. Moreover, it is important to find proper classical dynamics applicable not only to the $\mathbb Z_2$ spin variable but also to the binary (0 and 1) and multi-valued variables. Also, the algorithmic difference between our HQA (which preserves the adiabaticity from the beginning to the end) and SBM^23^ (which breaks the adiabaticity at the point of bifurcation) should be clarified to understand the role of classical adiabaticity. It is also important to find a mathematical theorem which can quantify how close our MD solver can approach to the ground state. With all these future works, our quantum–classical hybrid scheme provides a promising method to obtain efficient and precise solutions for optimization problems in science and technology.

## Methods

### Computational systems

For quantum annealing processor, we utilized the lower-noise D-Wave 2000Q quantum processor DW_2000Q_5 in our numerical experiments. The scheduling functions and the working graph of this processor is available in Ref.^25^. It enables us to embed the 48-node complete graph $K_{48}$ to this processor with the standard triangle clique embedding scheme (see e.g. Ref.^31^). Quantum annealing is conducted with chain_strength = 15, num_reads = 10,000, postprocess = ‘optimization’, and annealing_time = 20 [$\upmu$sec]. The chain_strength parameter is optimized with random instances associated with the Ising spin-glass. For classical computation, we utilized a system composed of Intel Xeon Platinum 8260 CPU @ 2.40 GHz (384 GB memory) and NVIDIA TeslaV100 GPU (32 GB memory). GPU acceleration is utilized in the case of MD calculations, for which parallel computation on GPU can be implemented in a straightforward manner.

### Scheduling functions for MD

We employ $\alpha (\tau ) = \alpha _f \bigl ( \tau + \rho _1 (1- \tau ) + \rho _2 \tau (\tau -1) \bigr )$ and $\beta (\tau ) = \beta _f \bigl ( \tau +\kappa _1 (1- \tau ) + \kappa _2 \tau (\tau -1 ) \bigr )$, with $(\alpha _f, \rho _1, \rho _2) = ( 0.008, 4, 3)$ and $(\beta _f , \kappa _1, \kappa _2)= (0.12, 0.05, 1)$. In early times when $\alpha (\tau ) \gg \beta (\tau )$, the flux variables $\{\varphi _i\}_{i=1}^N$ oscillate around $\varphi _i = 0$. This is a classical analogue of the initial quantum-superposition state of quantum annealing. If the motion of the flux variables is sufficiently faster than the evolution of scheduling functions, the system approaches adiabatically to the final state where most of the flux variables $\{\varphi _i\}_{i=1}^N$ tend to be localized.

### Leapfrog algorithm

The Hamilton equations in Eq. (4) for $i=1, \dots , N$ can be solved accurately by the leapfrog algorithm^32^. With a given initial condition at $\tau =0$, $\{\varphi _i(0), p_i(0)\}$, we integrate the Hamilton equations with the step size $\delta \tau$ being identified with *g* as follows:

$$\begin{aligned} {\left\{ \begin{array}{ll} p_i^{(m+ \frac{3}{2})} - p_i^{(m+\frac{1}{2})} &{}= \displaystyle - {\alpha }^{(m+1)} \left. \frac{\partial V (\varphi )}{\partial \varphi _i} \right| ^{(m+1)} - 2{\beta }^{(m+1)} \Bigl [ \frac{1}{2} \sum _{j=1}^N J_{ij} \varphi _j^{(m+1)} + h_i \bigl |\varphi _i^{(m+1)}\bigr |\Bigr ] , \\ \varphi _i^{(m+2)} - \varphi _i^{(m+1)} &{} = {\alpha }^{(m+\frac{3}{2})} p_i^{(m+\frac{3}{2})}, \end{array}\right. } \end{aligned}$$

together with the initial half step, $p_i^{(\frac{1}{2})} = p_i^{(0)} - \frac{ \alpha ^{(0)} }{2} \left. \frac{\partial V (\varphi )}{\partial \varphi _i} \right| ^{(0)} - \beta ^{(0)} \Bigl [ \frac{1}{2} \sum _{j=1}^N J_{ij} \varphi _j^{(0)} + h_i \bigl |\varphi _i^{(0)}\bigr |\Bigr ]$ and $\varphi _i^{(1)} = \varphi _i^{(0)} + \alpha ^{(\frac{1}{2})} p_i^{(\frac{1}{2})}$. Here *m* denotes the temporal step with $\tau =m \cdot \delta \tau$ ($m=0, 1, 2, \dots$). Also, we introduced an abbreviated notation, $f_i^{(m)} \equiv f_i(m \cdot \delta \tau )$ and $f_i^{(m+\frac{1}{2})} \equiv f_i((m +\frac{1}{2})\cdot \delta \tau )$ with $f=\varphi$, *p*, $\alpha$ and $\beta$. The leapfrog integrator has only $O((\delta \tau )^2)$ error and is essential for our MD evolution to be accurate enough. (If the Hamiltonian does not have explicit $\tau$-dependence which is not the case in the present situation, this integrator has nicer properties such as the time-reversibility and the symplectic property.)

### MAX-CUT and Ising spin-glass

For a given undirected graph ${\mathcal {G}}=({\mathcal {V}}, {\mathcal {E}})$ with an edge-weight $\{w_{ij}\}_{(ij)\in {\mathcal {E}}}$, the MAX-CUT is a problem of finding a partition of vertices, ${\mathcal {V}} = {\mathcal {V}}_+ \cup {\mathcal {V}}_-$ with ${\mathcal {V}}_+ \cap {\mathcal {V}}_- = \emptyset$, which maximizes the sum of $w_{ij}$ connecting the two sets, $C\equiv \sum _{i \in {\mathcal {V}}_+, j \in {\mathcal {V}}_-, (ij) \in {\mathcal {E}}} w_{ij}$. This can be mapped to the problem of maximizing $C(s) = \frac{1}{2}\sum _{(ij) \in {\mathcal {E}}} w_{ij} {(1 - s_i s_j)}$ with respect to the Ising spin variables $s_i = \pm 1$. One can rewrite *C*(*s*) in terms of the Ising spin-glass model ($J_{ij}=w_{ij}$, $h_i=0$) as $C(s)= - \frac{1}{2}{{\mathcal{H}}_{\mathrm{Ising}}(s)} + C_0$, with ${\mathcal{H}}_{\mathrm{Ising}}(s) = \frac{1}{2} \sum _{i\ne j} J_{ij} s_i s_j$ and $C_0 \equiv \frac{1}{4}\sum _{i\ne j} J_{ij}$. Minimizing the Ising energy ${\mathcal{H}}_{\mathrm{Ising}}(s)$ corresponds to maximizing the cut configuration. The instances of our experiment are given on the 2000-node complete graph $K_{2000}$ with randomly generated bimodal weights $J_{ij} = \pm 1$. Therefore, the constant $C_0$ follows the normal distribution with zero-mean for large *N*. The ground-state energy averaged over instances, $E^* \equiv \left\langle {\mathcal{H}}^{\mathrm{(min)}}_{\mathrm{Ising}}(s) \right\rangle$ for the corresponding spin-glass model has been discussed in Ref.^29^: The finite-size scaling implies $E^*/N^{\frac{3}{2}} \xrightarrow [N \rightarrow \infty ]{} e_0 + A/ N^{\omega }$. Here $e_0 = -0.7631667265(6)$ is the Parisi energy^33^, while $\omega =2/3$ and $A = 0.70(1)$ are a conjectured value and a fitted value, respectively, of the numerical data for finite *N*. Combining all, the estimated value of the maximum-cut $C^*$ on $K_{2000}$ reads $C^* \equiv -E^*/2 = 33933(4)$, which we refer in Fig. 6.

## Supplementary information

Supplementary Information.
