# Supplementary material for: Hybrid quantum annealing via molecular dynamics
Source: Sci Rep. 2021 Apr 19;11:8426. doi: 10.1038/s41598-021-87676-z (PMC8056001; doi:10.1038/s41598-021-87676-z)
Supplement: Supplementary file 1 — Supplementary Information. [file 41598_2021_87676_MOESM1_ESM.pdf]

Supplementary Information

Hybrid Quantum Annealing via Molecular Dynamics

Hirotaka Irie*, Haozhao Liang, Takumi Doi, Shinya Gongyo, and Tetsuo Hatsuda

* Correspondence to hirotaka.irie.j3a@jp.denso.com

Supplementary Note 1: Initial condition in HQA

In the main text, we studied the MAX-CUT problem on the 2000-node complete graph K_{2000}, averaged over 100 instances with a single initial condition, $\varphi_i(0) = 0$ and $p_i(0)$ taken randomly from ± 1. To check the initial-condition dependence, we consider MAX-CUT problem over a single instance with 100 initial conditions, $\varphi_i(0) = 0$ and a set of $p_i(0)$ taken randomly from ± 1.

Fig. S.1(a) shows the distribution of the maximum cut C for a single instance with 100 initial conditions by using the MD solver and the HQA(TS1000) solver. One finds that (i) the central value of the distribution increases as the MD steps increase (the same behavior as the case of the 100 instances with a single initial condition), (ii) the distribution becomes sharper as the MD steps increase, and (iii) HQA provides larger C with sharper distribution than those of MD, which becomes prominent for large MD steps. To highlights the feature (iii) in a magnified scale, we show in Fig. S.1(b) a comparison of the distributions of C among MD, HQA(DW48) and HQA(TS1000) after 500,000 MD steps.

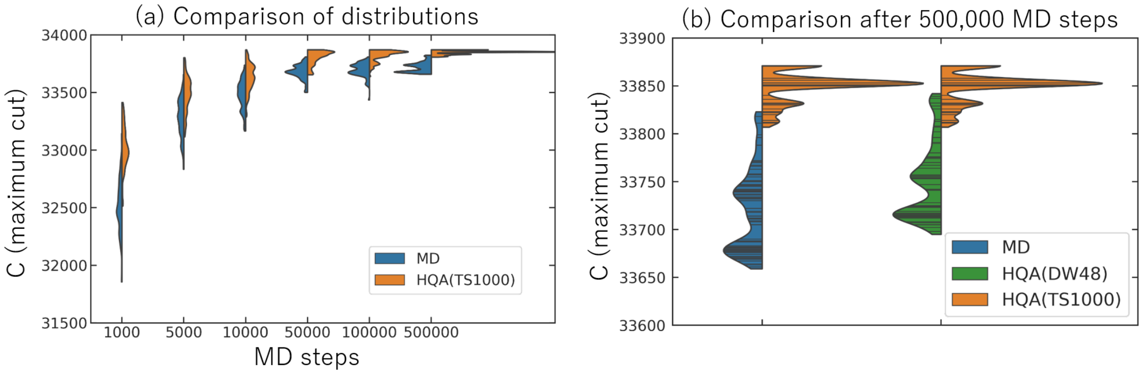

Figure S.1: (a) Distributions of C associated with 100 initial conditions for the MAX-CUT problem on K_{2000} by using the MD solver and the HQA(TS1000) solver. (b) Comparison of the distribution of C after $500,000$ MD steps among MD, HQA(DW48) and HQA(TS1000). The horizontal lines in the distributions represent data points of 100 initial conditions.

Supplementary Note 2: Adiabaticity in MD

We have taken specific forms of the scheduling functions $\alpha(\tau)$ and $\beta(\tau)$ for MD in the main text. In this note, we study the adiabaticity of the MD evolution by changing one of the parameters κ_2 in $\beta(\tau) = \beta_f\Big(\tau + \kappa_1(1-\tau) + \kappa_2\tau(\tau-1)\Big)$. Keeping $\beta_f = 0.12$ and $\kappa_1 = 0.05$ as default values in the main text, we change κ_2 within the interval $[-1, 1]$ from the default value $\kappa_2 = +1$. We do not change $\alpha(\tau)$ for simplicity. Shown in Fig. S.2(a) are typical three paths of $\beta(\tau)$ as a function of τ.

With the above scheduling functions, we solve the Ising spin-glass problem of $N = 10,000$ with a single instance and 10 initial conditions, and calculate the final values of the MD Hamiltonian at $\tau = 1$ for different MD steps. In Fig. S.2(b), $\mathcal{H}_{\rm MD}(\tau = 1)$ normalized by its best available value $\mathcal{H}_{\rm best}(\tau = 1)$ corresponding to $\kappa_2 = 1$ and $(\delta\tau)^{-1} = 500,000$ are plotted against κ_2. We find that (i) the value of $\mathcal{H}_{\rm MD}(\tau = 1)$ is nearly independent of κ_2 as long as the MD steps are large enough, indicating that the adiabatic evolution is at work in our MD, and (ii) the approach to $\mathcal{H}_{\rm best}(\tau = 1)$ is fastest for the default value $\kappa_2 = +1$ employed in the main text.

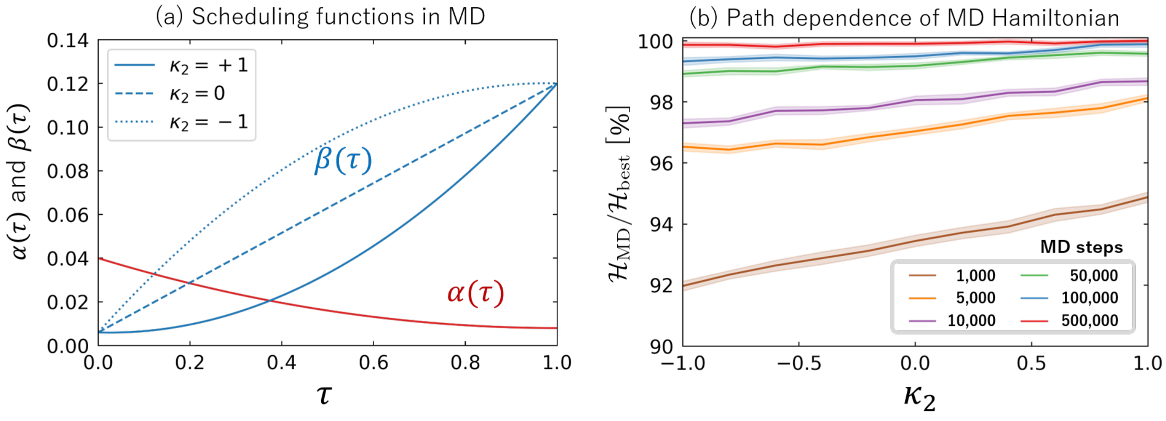

Figure S.2: (a) Typical three paths of thre scheduling function $\beta(\tau)$, parametrized in the interval $-1 \le \kappa_2 \le 1$. (b) The MD Hamiltonian at the end of the MD evolution $\mathcal{H}_{\rm MD}(\tau = 1)$ normalized by its best available value $\mathcal{H}_{\rm best}(\tau = 1)$ as a function of κ_2.
